# Emergent patterns of island biodiversity in the Anthropocene

**DOI:** 10.1126/sciadv.aee1788

**Published:** 2026-07-23

**Authors:** Thomas J. Matthews, Ferran Sayol, Filipa C. Soares, Julia H. Heinen, Osanna Chu, Natàlia Martínez-Rubio, Joseph P. Wayman, Thomas E. Martin, Roberto Rozzi, Céline Bellard, Tim M. Blackburn, Paulo A. V. Borges, Michael K. Borregaard, Juliano Sarmento Cabral, Pedro Cardoso, Bo Dalsgaard, Simone Fattorini, José María Fernández-Palacios, Matthew R. Helmus, Julian P. Hume, Michał T. Jezierski, Holger Kreft, Oriol Lapiedra, Mark V. Lomolino, Sandra Nogué, François Rigal, Ana M. C. Santos, Julian Schrader, Xingfeng Si, Jens-Christian Svenning, Kostas A. Triantis, Luis Valente, Alexandra A. E. van der Geer, Patrick Weigelt, Robert J. Whittaker, Søren Faurby

**Affiliations:** ^1^ School of Geography, Earth and Environmental Sciences (GEES) and Birmingham Institute of Forest Research, University of Birmingham, Birmingham B15 2TT, UK.; ^2^ CE3C – Centre for Ecology, Evolution and Environmental Changes/Azorean Biodiversity Group / CHANGE – Global Change and Sustainability Institute and Universidade dos Açores – Faculty of Agricultural Sciences and Environment, PT-9700-042 Angra do Heroísmo, Açores, Portugal.; ^3^ BETA Technological Centre - University of Vic - Central University of Catalunya (BETA- UVIC- UCC), 08500 Vic, Spain.; ^4^ BiBio Research Group, Natural Sciences Museum of Granollers, 08402 Granollers, Spain.; ^5^ CIBIO, Centro de Investigação em Biodiversidade e Recursos Genéticos, InBIO Laboratório Associado, Campus de Vairão, Universidade do Porto, 4485-661 Vairão, Portugal.; ^6^ CIBIO, Centro de Investigação em Biodiversidade e Recursos Genéticos, InBIO Laboratório Associado, Instituto Superior de Agronomia, Universidade de Lisboa, 1349-017 Lisboa, Portugal.; ^7^ BIOPOLIS Program in Genomics, Biodiversity and Land Planning, CIBIO, Campus de Vairão, 4485-661 Vairão, Portugal.; ^8^ Palaeogenomics and Bio-Archaeology Research Network, School of Archaeology, University of Oxford, Oxford OX1 3AN, UK.; ^9^ Herbarium, Royal Botanic Gardens, Kew, Richmond, Surrey TW9 3AB, UK.; ^10^ Centre for Ecological Research and Forestry Applications (CREAF), 08193 Catalonia, Spain.; ^11^ Operation Wallacea Ltd, Wallace House, Old Bolingbroke, Spilsby, Lincolnshire PE23 4EX, UK.; ^12^ Department of Biological Sciences, Simon Fraser University, Burnaby, BC, Canada.; ^13^ Zentralmagazin Naturwissenschaftlicher Sammlungen, Martin Luther University Halle-Wittenberg, 06108 Halle (Saale), Germany.; ^14^ Museum für Naturkunde – Leibniz-Institut für Evolutions- und Biodiversitätsforschung, 10115 Berlin, Germany.; ^15^ Université Paris-Saclay, CNRS, AgroParisTech, Ecologie Société Evolution, Gif-sur-Yvette, France.; ^16^ Centre for Biodiversity and Environment Research, University College London, Gower Street, London WC1E 6BT, UK.; ^17^ Institute of Zoology, Zoological Society of London, Regent’s Park, London NW1 4RY, UK.; ^18^ IUCN SSC Atlantic Islands Invertebrate Specialist Group, 9700-042 Angra do Heroísmo, Azores, Portugal.; ^19^ IUCN SSC Species Monitoring Specialist Group, 9700-042 Angra do Heroísmo, Azores, Portugal.; ^20^ Globe Institute, University of Copenhagen, Universitetsparken 15, 2100 Copenhagen, Denmark.; ^21^ School of Biosciences and Birmingham Institute of Forest Research, University of Birmingham, Birmingham B15 2TT, UK.; ^22^ Ecological Modelling, Bonner Institute for Organismal Biology - Department of Plant Biodiversity, University of Bonn, Venusbergweg 22, 53115 Bonn, Germany.; ^23^ Centre for Ecology, Evolution and Environmental Changes (CE3C), Global Change and Sustainability Institute (CHANGE), Faculty of Sciences, University of Lisbon, 1749-016 Campo Grande, Lisboa, Portugal.; ^24^ Section for Molecular Ecology and Evolution, Globe Institute, University of Copenhagen, 1353 Copenhagen, Denmark.; ^25^ Department of Life, Health and Environmental Sciences, University of L’Aquila, Via Vetoio, 67100 L’Aquila, Italy.; ^26^ Island Ecology and Biogeography Group, Instituto Universitario de Enfermedades Tropicales y Salud Pública de Canarias (IUETSPC), Universidad de La Laguna (ULL), 38200 La Laguna, Canary Islands, Spain.; ^27^ Department of Biology, Temple University, 1925 N. 12th Street, Philadelphia, PA 19122, USA.; ^28^ Bird Group, Natural History Museum, Akeman Street, Tring HP23 6AP, UK.; ^29^ Department of Biodiversity, Macroecology and Biogeography, University of Göttingen, Büsgenweg 1, 37077 Göttingen, Germany.; ^30^ Centre of Biodiversity and Sustainable Land Use (CBL), University of Göttingen, Büsgenweg 1, 37077 Göttingen, Germany.; ^31^ Campus Institute Data Science (CIDAS), University of Göttingen, Goldschmidtstraße 1, 37077 Göttingen, Germany.; ^32^ College of Environmental Science and Forestry, State University of New York, Syracuse, NY 13210, USA.; ^33^ Departament de Biologia Vegetal, Biologia Animal i d’Ecologia (BABVE), Universitat Autònoma de Barcelona, 08193 Belaterra, Catalonia, Spain.; ^34^ Université de Pau et des Pays de l’Adour, CNRS, IPREM, IMT Mines ALES, Pau, France.; ^35^ Terrestrial Ecology Group (TEG-UAM), Departamento de Ecología, Universidad Autónoma de Madrid, Calle Darwin 2, 28049 Madrid, Spain.; ^36^ Centro de Investigación en Biodiversidad y Cambio Global (CIBC-UAM), Universidad Autónoma de Madrid, Calle Darwin 2, 28049 Madrid, Spain.; ^37^ School of Natural Sciences, Macquarie University, NSW, Australia.; ^38^ Center for Global Change and Ecological Forecasting, Zhejiang Zhoushan Island Ecosystem Observation and Research Station, Zhejiang Tiantong Forest Ecosystem National Observation and Research Station, Institute of Eco-Chongming, School of Ecological and Environmental Sciences, East China Normal University, Shanghai, China.; ^39^ Center for Ecological Dynamics in a Novel Biosphere (ECONOVO), Department of Biology, Aarhus University, Ny Munkegade 114, DK-8000 Aarhus C, Denmark.; ^40^ Department of Ecology and Taxonomy, Faculty of Biology, National and Kapodistrian University of Athens, Athens GR-15784, Greece.; ^41^ Naturalis Biodiversity Center, 2333CR Leiden, Netherlands.; ^42^ Groningen Institute for Evolutionary Life Sciences, University of Groningen, 9747AG Groningen, Netherlands.; ^43^ Department of Environmental Science, Radboud Institute for Biological and Environmental Sciences (RIBES), Radboud University, Heyendaalseweg 135, 6525AJ Nijmegen, Netherlands.; ^44^ School of Geography and the Environment, and St Edmund Hall, University of Oxford, Oxford OX14AR, UK.; ^45^ Center for Macroecology, Evolution and Climate, Globe Institute, University of Copenhagen, Copenhagen 1350, Denmark.; ^46^ Department of Biological and Environmental Sciences, University of Gothenburg, Box 461, SE 40530 Gothenburg, Sweden.; ^47^ Gothenburg Global Biodiversity Centre, Box 461, SE 40530, Gothenburg, Sweden.

## Abstract

Islands have long been foundational to the development of ecological and evolutionary theory, serving as model systems for studying patterns of biodiversity. However, pervasive anthropogenic impacts—particularly species extinctions and introductions—have substantially altered island ecosystems, complicating inferences drawn from contemporary data. Here, we review how these human-driven changes have reshaped key biodiversity patterns on islands, including patterns of species richness, community composition, evolution, and species interactions. We show that many current ecological and evolutionary patterns observed on islands reflect not just natural processes but also a legacy of human-induced change spanning millennia. Approaches that better incorporate paleoecological evidence, along with improved island biodiversity databases and modeling techniques, can help better reconstruct prehuman biodiversity baselines on islands.

## Teaser

Human impacts substantially affect our ability to detect and understand natural biodiversity patterns.

## INTRODUCTION

A core aim of biodiversity research is the identification of general patterns and processes at different spatial and temporal scales. Islands are model systems in this regard, with island research being fundamental to the development of a wide range of ecological and evolutionary theories, including the equilibrium theory of island biogeography and the theory of evolution via natural selection (*1*–*4*). However, human activities have substantially altered the biota of many islands around the world through both the extinction of island species and the introduction of large numbers of non-native species (*5*–*7*). Species introductions and extinctions typically commenced at the point of first human colonization, meaning that anthropogenically driven changes in insular species composition started thousands of years ago in many archipelagos (*8*–*11*).

Insular endemic species account for around 75% of International Union for Conservation of Nature (IUCN)-documented global extinctions (*12*, *13*). In addition, islands are known to have suffered numerous anthropogenic global extinctions before the 1500–common era (CE) baseline used by IUCN (*14*, *15*), and there have also been many additional island-level extirpations: Some islands have lost almost all of their native species from certain taxa. While extinction has always been a part of island life, the anthropogenic insular extinction crisis is unique on a ≥30-million-year timescale given the (i) global extent of island extinction events spanning multiple taxa and archipelagos (*13*, *16*–*18*), (ii) strong trait selectivity of extinctions [e.g., the disproportionate loss of larger-bodied species; (*19*, *20*)], and (iii) extent to which extinction rates exceed estimated background extinction rates, in many cases by an order of magnitude or more (*14*, *15*, *21*, *22*). We use the term “Anthropocene” simply as shorthand to refer to the general period during which humans have affected biodiversity on islands—the starting point of which will differ between islands—rather than as a formally defined period of time with a specific start point.

Islands are also hotspots of non-native species introductions (*23*–*26*), with non-native species often being a driver of extinctions (*13*). The numbers involved are often substantial. For example, approximately 1500 introduced terrestrial and marine species are known to have become established on the Galápagos Islands (*27*), while the Hawaiian flora now includes more than 1400 naturalized non-native species, roughly the same number as in the original flora (*28*).

Several decades ago, mounting evidence of the extent of prehistoric insular extinctions on Hawai’i led to warnings that “…biogeographical inferences about natural processes based only on historically known taxa may be misleading or incorrect” [(*29*), p. 633]. However, despite extensive subsequent research on the human transformation of island environments (*5*, *6*, *9*, *30*, *31*), there is still an often unrecognized but potentially substantial incongruity between the role of islands in advancing our understanding of “natural” biodiversity patterns and processes and the extensive alteration they have undergone because of human impacts. In recognition of the botanist Joseph Dalton Hooker (1817–1911), this knowledge gap has been termed the Hookerian shortfall: the shortfall in our understanding of the extent to which observed biodiversity patterns are natural or shaped by human influence (*32*, *33*).

Here, we assess and exemplify anthropogenic impacts on biodiversity patterns on islands, including patterns of (i) species richness, (ii) community composition, (iii) evolution, and (iv) species interactions. We place a particular focus on the extent to which omitting species driven extinct by humans, as well as failing to account for introduced species, may result in biased patterns and thus skew our understanding of the underlying natural processes. Our focus is on marine islands, particularly oceanic islands (i.e., islands of volcanic or coralline formation that have never been connected to mainland areas) and ancient continental fragments (e.g., Madagascar) given: (i) their historical and contemporary importance in the development of island biology theory; (ii) that they represent discrete, clearly demarcated geographic units; and (iii) that most recorded anthropogenic extinctions have been from such islands.

## ISLAND DIVERSITY RELATIONSHIPS

Given its role as the basic currency of island biodiversity research, we begin with island species richness. For a wide range of taxa, native richness has declined on many islands globally because of anthropogenic extinctions and extirpations (*6*, *13*, *31*, *34*–*36*). These declines have in many cases been considerable. To take three examples, Christmas Island (Indian Ocean) has lost 80% of its native mammals and lizards (*37*), Rodrigues in the Mascarenes has lost 100% of its endemic reptiles, and the Hawaiian Islands have lost approximately 70% of their native terrestrial birds (*38*) and 65 to 90% of their land snail species (*39*). Notwithstanding such losses, many islands have gained so many species through anthropogenic introductions that their current species richness exceeds that existing before human colonization (*23*, *36*, *40*, *41*).

Anthropogenic extinctions and introductions are typically nonrandom with respect to species’ traits and phylogeny (*19*, *20*, *24*): Traits once beneficial in prehuman insular environments (e.g., flightlessness in birds) may have become maladaptive in human-modified environments, while species from particular clades, and with specific sets of traits, are disproportionately introduced (e.g., livestock and commensal rodents). Hence, changes in richness have often been accompanied by changes in functional diversity (the diversity of species traits) and phylogenetic diversity (the breadth of evolutionary history represented by a set of species) (*41*–*44*). For example, bird extinctions have often resulted in substantial declines in island functional diversity due to anthropogenic drivers preferentially targeting relatively functionally unique species; for example, larger and flightless birds are more vulnerable to human hunting and to introduced predators (*15*, *19*, *34*). On the other hand, insular avian introductions tend to involve a small subset of families (*45*), resulting in relatively small compensatory increases in functional diversity (*46*, *47*) or “false compensation” via increases in previously unoccupied areas of functional and phylogenetic space (*41*).

**Fig. 1.**
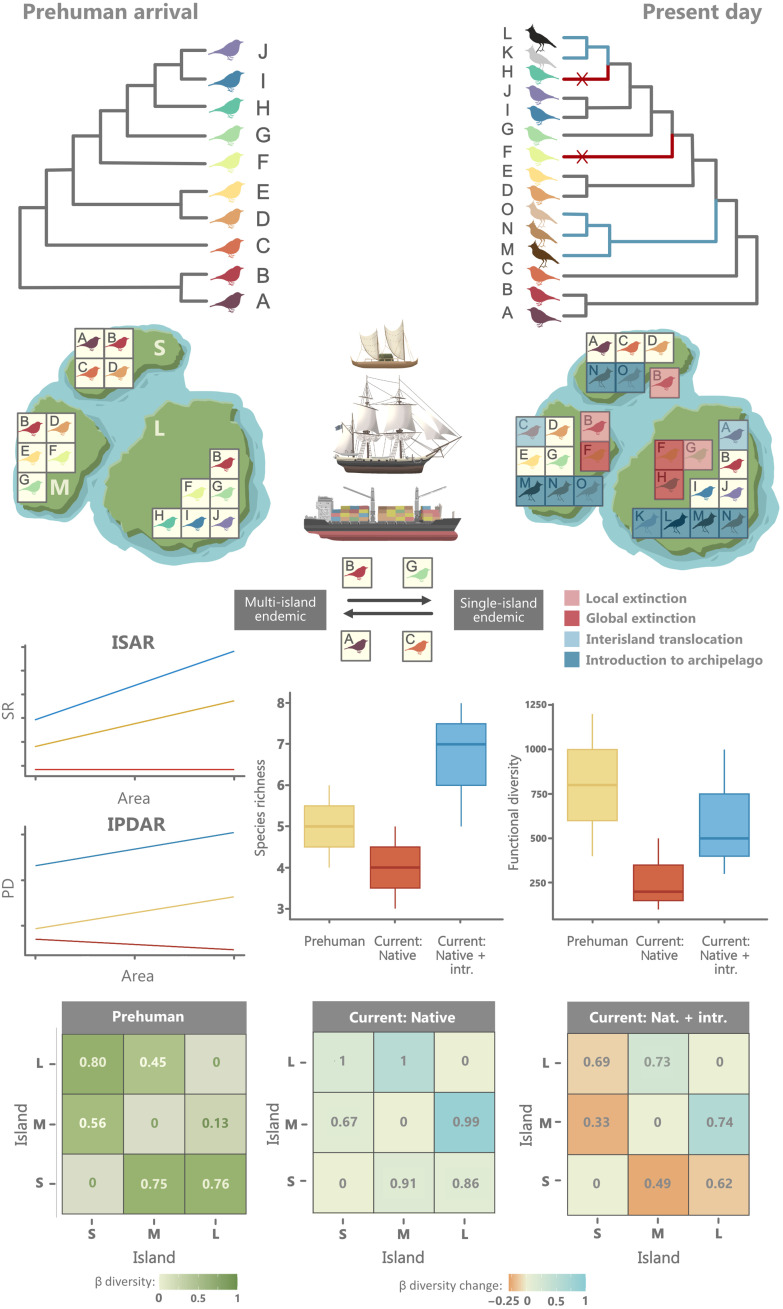
The effect of species extinctions and introductions on island biodiversity patterns. The second row shows an archipelago comprising three islands of different sizes [small (S), medium (M) and large (L)], with prehuman arrival (left) and present-day (right) composition both shown. The species present on the islands (species A to J) before human arrival are shown, with the phylogeny (first row) depicting the evolutionary relationships between species (colors represent different species). Species differ in their functional traits [beak length and mass values randomly sampled from AVONET; (*155*)], but this is not shown. Following human arrival, some species have (i) gone globally extinct (red branches in the phylogeny), (ii) gone locally extinct (i.e., extirpated from one island but remain in the archipelago), (iii) been introduced to the archipelago from elsewhere (blue branches in the phylogeny), or (iv) been translocated to one island from another. The third and fourth rows show how these assemblage changes affect various biodiversity metrics and patterns: the ISAR and phylogenetic diversity–area (IPDAR) relationship, functional diversity and species richness (boxplots show the average and spread of diversity values across islands), and differences in species and functional trait composition between islands (i.e., taxonomic and functional β diversity). In regard to the latter, a pairwise dissimilarity matrix based on the archipelago composition data is shown, depicting pairwise β diversity measured using Sørensen’s dissimilarity (larger values = larger difference in composition): The top triangle shows pairwise taxonomic β diversity and the lower triangle functional β diversity. The color scale in the present-day dissimilarity matrices (bottom middle and right) shows changes in Sørensen’s dissimilarity relative to the respective prehuman value [red, decreases in Sørensen’s dissimilarity (homogenization); blue, increased dissimilarity (differentiation)]. The data used in the line graphs, boxplots, and dissimilarity matrices represent the island communities, while the boxplots use some simulated data for illustrative purposes.

Often, island biologists calculate species functional and phylogenetic diversity as the first step toward investigating a broader set of emergent diversity patterns (Fig. 1). One such pattern is the island diversity–area relationship (IDAR)—a pattern that describes how some measure of island diversity scales with island area. Typically, these relationships are well fitted by a power model (*4*, *6*) which, when both quantities are logarithmically transformed, becomes a linear model with two parameters: intercept (*c*) and slope (*z*). However, other models (e.g., sigmoidal models) have been found to provide better fits than the power model in some cases (*6*). IDARs include the foundational island species–area relationship (ISAR), where the diversity measure is species richness—a pattern that holds both theoretical and applied importance in biodiversity research (*2*, *6*, *48*, *49*). Some studies have argued that extinctions should result in steeper IDAR slopes if analyses are restricted to islands above a certain size [e.g., (*50*)]. However, an evaluation of birds on islands in 10 archipelagos found that extinctions tended to flatten IDAR power model slopes, while introductions tended to steepen them [(*51*); see also (*5*)], although there is considerable variation around this general trend, likely due in part to variation in the amount of paleoecological work undertaken across islands (*31*). Some of these shifts in slope (i.e., *z*) are relatively substantial; for example, for the ISAR, known extinctions and extirpations across the Marianas have resulted in a 70% decrease in slope (from *z* = 0.20 to 0.06) relative to the prehuman ISAR. This trend likely reflects two factors: (i) Larger islands have higher speciation rates (*52*, *53*), producing more specialized single-island endemics vulnerable to anthropogenic impacts (*6*), and (ii) larger islands often host more people, leading to greater impacts from land use change, trade, and introduced species (*23*, *54*). While this study (*51*) focused specifically on the power model, we note that the general finding—that extinctions and introductions will alter IDAR model parameter values—will hold irrespective of the mathematical model used, given that the underlying island diversity values are being altered.

The form of IDARs has also changed. For example, the prehuman ISAR of Caribbean *Anolis* lizards was biphasic, with an increase in ISAR slope above a threshold island size where speciation starts to become an important assembly process (*53*), but recent species introductions, which have disproportionately elevated the richness of previously species-poor (particularly midsized and isolated) islands, have linearized this relationship (*5*).

Another fundamental island biodiversity pattern that has been significantly reshaped by anthropogenic extinctions and introductions is the tendency for species richness to decrease with increasing island isolation (*2*). Several species groups (e.g., mammals, plants, reptiles, and ants) have been found to now display positive non-native species richness–isolation relationships (*5*, *54*, *55*). Economic factors such as international trade routes, rather than simply distance from source pools, largely determine introduction rates in the historic period, and this colonization pressure is the strongest determinant of non-native species richness (*56*). The number of species introduced has been shown to explain the species–isolation relationship in non-native birds (*57*), although a lack of biotic resistance from depauperate native assemblages of highly isolated islands may perhaps have a role for other taxa (*55*).

Changes to island species richness patterns also involve changes to individual species distribution patterns. Numerous extant species are currently recognized as single-island endemics [e.g., there are estimated to be >40,000 single-island endemic plants (*58*)], but evidence suggests that in many cases such species were originally (often much) more broadly distributed, being natural multi-island endemics before human extirpations. These “Anthropocene single-island endemics” include, for example, the critically endangered Cuban crocodile (*Crocodylus rhombifer*), which is now endemic to Cuba but was found on numerous Caribbean islands before human arrival (*59*). The opposite situation—whereby humans have introduced single-island endemics to multiple islands in the same region—has also been observed. Examples include numerous mammal and herptile species in Melanesia (*8*, *60*), with several of these interisland translocation events believed to have occurred more than 10 thousand years ago, making them challenging to detect. Even classifying species as naturally endemic to islands can be challenging, as anthropogenic extirpations of mainland populations may create “artificial island endemics” (*18*), such as the Tasmanian devil (*Sarcophilus harrisii*), now endemic to the island of Tasmania but previously distributed across much of mainland Australia (*61*).

For species such as the aforementioned Cuban crocodile, detailed ecological, ethnological and paleoecological research means that we have a good understanding of their prehuman-arrival insular distributions and can thus update their contemporary endemism status accordingly. However, there are several biases involved in island paleoecological research, including geographic spatial biases (i.e., more paleoecological work on certain islands than others) and taxonomic biases (i.e., certain groups are underrepresented in the fossil record) (*31*, *62*, *63*). Thus, it is likely that there are numerous unrecorded extinction, extirpation, and translocation events—particularly those that occurred before the historic period—that we cannot account for in analyses (*8*, *14*, *60*). Even within birds, perhaps the best studied taxon, there are estimated to be more than 750 unrecorded anthropogenic island endemic extinctions globally, an ∼150% increase on the number of known island endemic extinctions (*14*). This is concerning not just from a conservation but also a theoretical point of view as several island ecological and evolutionary theories and models make specific predictions about metrics such as the number of endemic species and speciation rates on islands, which could be biased if the number of unrecorded events is nontrivial.

## SHIFTING SPECIES COMPOSITION ON ISLANDS

**Fig. 2.**
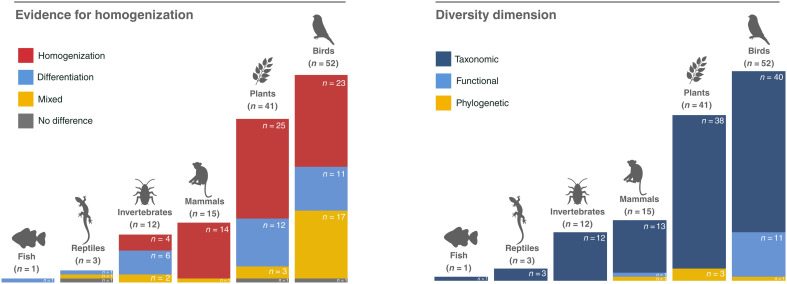
Summary of the results of studies of island biotic homogenization. Homogenization is defined here as increased compositional similarity in some measure of diversity through time. Study results are split across taxa. The left panel shows the main study finding, and the right panel shows the diversity dimension analyzed (taxonomic, functional, or phylogenetic). Data were primarily compiled from a literature search conducted in Web of Science on 7 June 2025 using the search terms: AB = [Island* AND (homogenization OR homogenisation)] OR TI = [Island* AND (homogenization OR homogenisation)]. AB, abstract; TI, title. Further searches were subsequently undertaken using the keywords “beta-diversity” (and variants thereof) and “differentiation.” Studies were included if they assessed change in compositional similarity across true islands within an archipelago, across islands or archipelagos within a larger region, or at the global scale, but not within an individual island. However, for a given study, we took the results from the smallest unit of analysis (e.g., the within archipelago scale). If a given study analyzed compositional similarity within multiple archipelagos [e.g., (*70*)] or for multiple taxa or diversity dimensions in a single archipelago, we counted each of these as distinct results [i.e., the unit of analysis was taken to be a given taxon-diversity dimension-archipelago (or broader spatial scale) combination]. A total of 24 studies were deemed to be suitable for inclusion, comprising 124 individual analysis results. Some results were obtained from the source paper authors. More information and the underlying summary data are available on GitHub (“txm676/AnthroComp”).

Because of the counterbalancing effects of anthropogenic introductions and extinctions, the net change in insular richness may be of lesser magnitude than the changes in species composition per island (*9*, *64**–**66*). Changes in species composition have been observed both within and between archipelagos and across multiple dimensions of diversity (i.e., taxonomic, functional, and phylogenetic) (Fig. 2). These compositional shifts have often resulted in biotic homogenization—the process of increasing compositional similarity between islands (and between islands and mainland areas) (Fig. 2). Homogenization is theorized to be primarily driven by the extinction of single-island endemics—species that are frequently also functionally unique (*15*)—combined with the introduction of widespread species that are often functionally similar to one another (*17*, *36*). However, the level of homogenization varies across archipelagos and taxa and is influenced by factors such as taxon-specific traits (e.g., dispersal capacity), evolutionary history, archipelago configuration, environmental heterogeneity across spatial scales, and the amount of human impact (*66*). In regard to introduced species, their individual contribution to changing species composition patterns will depend on how many islands within an archipelago they are introduced to and how this number changes through time (*67*).

Island homogenization analyses have focused on various taxa, particularly plants and birds. In respect to plants, the most commonly reported trend is one of decreasing floristic distinctiveness (i.e., increasing compositional similarity) over time as a result of human activities (Fig. 2) (*11*, *68*). For example, the introduction of non-native species has led to large decreases in distinctiveness between historic and contemporary floras across Atlantic and Pacific archipelagos (*64*), as well as among islands in the floral hotspot of Malesia (*69*). Floristic homogenization typically started soon after human colonization of islands, in some cases thousands of years ago (*11*, *69*), and thus for many islands, reconstructing prehuman island floras is not straightforward. For animal taxa, studies of birds (*66*, *70*, *71*), arthropods (*72*, *73*), and mammals (*36*, *65*) also report insular biotic homogenization following human colonization in several cases (Fig. 2).

Although homogenization is the most commonly reported outcome of humanity’s impact, there is also evidence of increasing biotic differentiation (i.e., reduced compositional similarity between islands) over time in several cases [e.g., (*74*); Fig. 2]. Increasing differentiation is theorized to occur when an environmental change driver exerts uneven impacts on different islands, generating divergent trajectories in community composition between islands. Adding nuance, human activities can simultaneously lead to homogenization in one dimension of diversity but differentiation in another. For example, a recent study on birds found that anthropogenic extinctions and introductions had resulted in some island assemblages of the Mascarenes becoming more similar to others in the same archipelago in terms of species composition but more dissimilar in terms of functional trait composition, while several islands in the Azores and Canary Islands exhibited the converse patterns (*70*). It is also the case that different patterns of compositional change can be observed at different spatial scales. For example, a simultaneous increase in compositional similarity among islands within an archipelago, coupled with increasing dissimilarity between those islands and islands in other archipelagos [e.g., as has been observed for birds on the Hawaiian Islands compared to other islands in the Pacific; (*71*)]. At very large spatial scales (e.g., comparisons of islands at the global scale), the typical pattern is seemingly one of homogenization (*65*).

If unaccounted for in analyses, anthropogenic shifts in species composition also have the potential to affect our understanding of a range of island assembly processes, including immigration and habitat filters, adaptive and nonadaptive radiation, the role of historical contingency and priority effects, and interspecific interactions (*6*, *31*, *75*). As one example, including extinct species in analyses of the Hawaiian avifauna reveals that many more species successfully colonized the archipelago and gave rise to endemic lineages than is evident from the extant avifauna alone (*30*). However, this effect was found to vary across guilds. Specifically, the number of successful colonization events is underestimated by at least 45% for waterbirds and 75% for raptors (*30*), while the inferred pattern for passerines remains largely unchanged despite the large number of passerine extinction events (*38*). Thus, extinctions have obscured the success of waterbirds and raptors in naturally colonizing this remote archipelago.

The near complete loss of raptors on the Hawaiian Islands—only a single native raptor species remains and only on the island of Hawai’i—may have also created a misguided perception of the islands being relatively predator-free before human colonization (*34*, *38*). This assumption is frequently made in island biology (see discussion in “Human influences on island evolutionary patterns” below). However, anthropogenic extinction of avian predators has been detected across a diversity of archipelagos and taxa, and thus the extent to which this assumption has been shaped by human impacts is an open question. For example, at least 59 island endemic birds of prey (orders Accipitriformes, Cathartiformes, Falconiformes, and Strigiformes) are now known to have been driven globally extinct, including species from the Caribbean, Rapa Nui, New Zealand, Madagascar, New Caledonia, the Mascarenes, and several islands in Macaronesia, the Bismarck Archipelago, and the Mediterranean (*10*). These losses are in addition to the numerous cases of island-level raptor extirpations [e.g., (*74*)].

The nonrandom, trait-selective nature of anthropogenic extinctions and introductions (*20*, *36*) has also markedly altered the functional composition of many islands (*41*, *46*, *47*, *76*) and will likely continue to do so going forward (*77*). Across several insular taxa—including birds, mammals, and beetles—larger-bodied species are more likely to have gone globally or locally extinct (*20**,* *78**–**80*), these being replaced by non-native species that are often relatively smaller-bodied (*81*, *82*). These opposing dynamics have resulted in often-substantial shifts in the body-size distribution of species on islands (*19*). For example, anthropogenic extinctions have led to a 27% reduction in the median body mass and a 98% reduction in the SD of body mass among island-endemic birds globally (*15*). Such shifts in individual traits underlie changes in metrics of multitrait functional diversity and composition, which are often used to infer the processes responsible for community assembly on islands. For instance, the isolation of, and environmental conditions on, islands are theorized to introduce immigration and environmental filters to successful colonization (*6*). According to theory, these filters limit the combinations of functional traits possessed by species in island communities, resulting in lower functional and phylogenetic diversity than expected (i.e., a “clustered” assembly pattern), depending on the null model and source pool used. However, work on island birds and mammals [e.g., (*42*, *74*, *83*, *84*)] has shown that anthropogenic extinctions and extirpations have often increased the amount of functional and phylogenetic clustering, likely due to extinctions disproportionately involving the removal of the more functionally unique species [i.e., an anthropogenic (dis)assembly filter (*15*)]. Thus, analyzing assembly patterns using contemporary island composition data could result in incorrect inferences regarding the importance of natural filters of island assembly.

## HUMAN INFLUENCES ON ISLAND EVOLUTIONARY PATTERNS

The biases associated with anthropogenic extinctions and introductions also complicate our understanding of evolutionary patterns on islands [e.g., (*32*, *85*)]. The clearest examples are found among species exhibiting traits typical of island syndromes [reviewed in (*6*)], which can be defined as “widespread patterns of morphological, behavioral, and demographic similarity in island-dwelling species, likely driven by convergent evolution and ecological filtering” (*86*). For example, several vertebrate taxa exhibit evolutionary shifts in body size on islands, with large-bodied species evolving smaller sizes and small-bodied species evolving larger sizes [i.e., the “island rule”; (*6*, *20*, *78*, *85*)], with the largest shifts seen in species of the most extreme size, the longest residence in isolation, and those inhabiting islands lacking competitors and terrestrial predators. However, at least among mammals, species with the greatest shifts in body size have been disproportionately driven to extinction, reducing our ability to detect the generality and intensity of the island rule based on extant species alone (*20*, *85*). Flightless birds are likewise overrepresented among extinct species (*10*, *87*), leading to a significant underestimation of the evolution of flightlessness—a common island syndrome in birds—when analyses are limited to extant species (*87*).

Behavioral and physiological shifts (*88*, *89*) may also be obscured by human impacts. For example, reduced terrestrial predator avoidance behavior may be both an island syndrome and have facilitated the extinction of several island endemics (*90*). As a result, comparative studies necessary to determine whether reduced predator avoidance is truly an island syndrome may, if based on data of extant species only, underestimate its prevalence. Similarly, slower metabolic rates (associated with slower life histories) in tetrapods are thought to be an island syndrome also associated with greater extinction risk (*89*), but the filtering effect of the latter makes it difficult to prove the former. While metabolic rates cannot be directly measured for extinct species, fossils do preserve indicators of such slower life histories (e.g., lower bone metabolism, longer lifespans, and delayed reproduction) (*91*, *92*).

The clearest evidence of anthropogenic activities confounding evidence of island syndromes is for tetrapods, particularly mammals and birds (*20*, *85*, *87*). However, this may simply reflect data availability, and there are other groups—largely lacking in fossil data—for which we can predict the presence of biased evolutionary patterns. For example, there are several proposed island syndromes associated with vascular plants, including secondary woodiness, loss of dispersibility, and the reduction in antiherbivore defenses (*6*, *93*). However, anthropogenic alteration of island ecosystems may have significantly affected particular syndromes; for instance, the introduction of non-native herbivores (*94*) means that certain adaptations (e.g., towards reduced herbivory) may no longer be beneficial (*95*). There is clear evidence of strong declines and extinctions in many island plant lineages (*96*). These extinctions may disproportionately involve species with certain island syndromes and, with many plant extinctions likely unrecorded (*97*), it is possible—although difficult to prove—that certain island syndromes in plants were more prevalent than they appear today.

Beyond these species-level traits, a range of other island evolutionary patterns have been modified by anthropogenic activities. For instance, with the exception of a single surviving species, an entire evolutionary radiation of helicinid snails on the Gambier Islands, including eight species on a single island, went extinct as a result of habitat loss and degradation before being scientifically described (*98*). It is only because snails leave behind shells that it was possible to identify this radiation postextinction, raising the possibility that many other invertebrate extinctions occurred on the islands without leaving a trace (*98*). To take another example, ignoring anthropogenic extinctions of noctilionoid bats in the Greater Antilles—a system where possibly over a third of species have been driven extinct—leads to an underestimation of colonization, speciation and turnover rates, as well as biased conclusions regarding whether island assemblages have reached equilibrium richness over evolutionary timescales (*99*). Anthropogenic activities may even lead to shifts in macroevolutionary equilibrium conditions and carrying capacities (*100*) on islands by (i) increasing the colonization rate (e.g., through introductions), (ii) raising the extinction rate (e.g., via ecosystem changes and hunting), and (iii) altering estimated speciation rates (e.g., by providing hybridization opportunities). Examples include the island fox of the California Channel Islands [*Urocyon littoralis* (*101*)], which evolved from mainland ancestors introduced by humans; the now-extinct Falkland wolf [*Dusicyon australis* (*102*)], whose ancestors may also have been introduced to the archipelago; and the York groundsel (*Senecio eboracensis*), a recently evolved micro-endemic species in Great Britain that arose as a hybrid between a native and a non-native ragwort species (*103*).

Island extirpations also affect our understanding of speciation drivers. For example, island area is an important correlate of speciation rate, with both theoretical and empirical studies arguing for a minimum island size below which cladogenesis is not detectable (*53*). However, in many cases, the relationship between island area and in situ speciation rate has been modified by anthropogenic extinctions and changes to species distributions. For example, on the basis of current distribution data for insular mammals, the smallest island globally with evidence of cladogenesis is Mindoro (∼9750 km^2^) for small mammals (rodents of the genus *Apomys*) and Sulawesi (∼180,700 km^2^) for large mammals (e.g., primates and water buffalo) (*104*). However, until the species involved went extinct, with human activities likely contributing to at least some of the extinctions, there was a radiation of large mammals (deer of the genus *Candiacervus*) on the much smaller island of Crete (∼8500 km^2^) which gave rise to an estimated eight species, although it is not known with certainty how many of these species coexisted at any one time (*105*).

Human activities have also created opportunities to study insular evolution over short timescales. Such opportunities include (i) the rapid adaptation of non-native species to novel island environments, such as body size shifts in non-native birds and mammals consistent with the island rule (*106*, *107*), and (ii) trait evolution in native species following anthropogenic impacts, such as predator introductions that have led to behavioral changes in native species and the partial reversal of some island syndromes. These behavioral changes include increasing nest height in Hawaiian birds (*108*) and an increase in escape distances in Galápagos iguanas (*109*). In addition, there is evidence that some island giants have experienced body size reduction, likely in response to human impacts, as has tentatively been observed in the giant lizards of the Canary Islands (*110*).

**Fig. 3.**
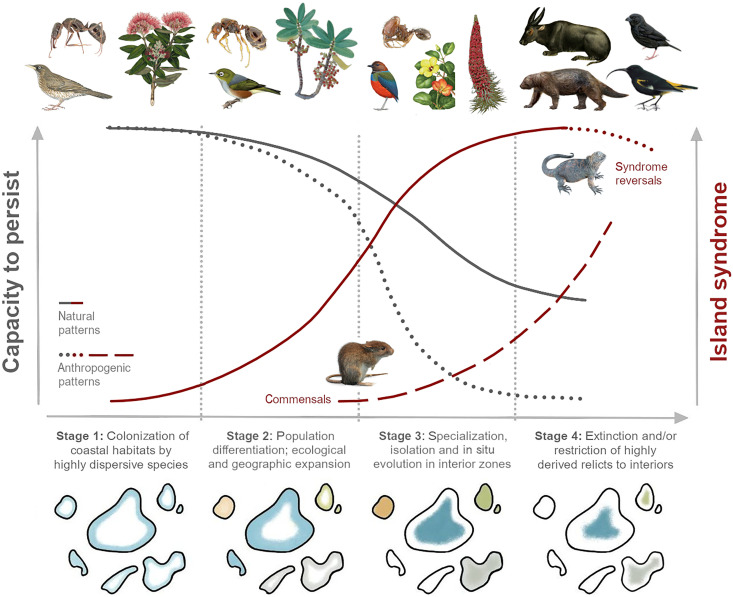
Anthropogenic influences on island syndromes and the capacity of species to persist through the different stages of the taxon cycle. The *x*-axis summarizes changes occurring during the expansion and contraction phases of the taxon cycle (colored shading in the islands along the bottom represents areas occupied by different species) and includes examples of species at different taxon cycle stages (top). Stage 1 (highly dispersive species): the pearly-eyed thrasher [*Margarops fuscatus*; Courtesy of Cornell Lab of Ornithology | (illustrated by I. Lewington) (https://doi.org/10.2173/bow) (*156*); CC BY-NC 4.0], the ant *Odontomachus simillimus* [by E. Prado, specimen CASENT0188531, from www.antweb.org (*157*), licensed under CC BY 4.0], and the pōhutukawa [*Metrosideros excelsa*; adapted from (*158*)]. Stage 2: the silvereye [*Zosterops lateralis*; Courtesy of Cornell Lab of Ornithology | (illustrated by D. Pratt) (https://doi.org/10.2173/bow) (*156*); CC BY-NC 4.0], the ant *Pheidole knowlesi* [by E.M. Sarnat, specimen CASENT0171042, from www.antweb.org (*159*); CC BY 4.0], and the ‘akoko spurge [*Euphorbia celastroides*; (*160*); CC BY-SA 4.0]. Stage 3: the Philippine pitta [*Erythropitta erythrogaster*; Courtesy of Cornell Lab of Ornithology | (illustrated by C. Rose) (https://doi.org/10.2173/bow) (*156*); CC BY-NC 4.0], the ant *Pheidole roosevelti* [(*161*); CC BY-SA 4.0] the dwarf mahoe [*Hibiscus glaber*; (*163*); used with permission of M. Yamanaka and Kew Gardens, copyright M. Yamanaka], and the red bugloss [Echium wildpretii; (*162*); CC BY-NC-SA 4.0]. Stage 4 (late taxon-cycle endemics): the tamaraw [*B. mindorensis*; adapted from (*164*)], the Cuban giant ground sloth [*Megalocnus rodens*; (*165*); used with permission of R. Uchytel], the Saint Lucia black finch [*Melanospiza richardsoni*; Courtesy of Cornell Lab of Ornithology | (illustrated by B. Small) (https://doi.org/10.2173/bow) (*156*); CC BY-NC 4.0], and the Hawai‘i mamo [*Drepanis pacifica*; Courtesy of Cornell Lab of Ornithology | (illustrated by R. Hathway) (https://doi.org/10.2173/bow) (*156*); CC BY-NC 4.0]. The inclusion of mammals as examples of stage 4 is speculative, but the taxa included are clear examples of island syndromes. Solid lines represent natural patterns: the development of the island syndrome in an island lineage (red) and the consequent decreased capacity to persist (gray). Dotted and dashed lines represent anthropogenic patterns: island syndrome reversals (red), such as behavioral adjustments to non-native predators in the Galápagos marine iguana [*Amblyrhynchus cristatus*; (*166*); used with permission of A. Arteaga], and the increase in vulnerability to extinction of late-taxon cycle species (gray). Also shown is an anthropogenic taxon cycle for introduced commensals [dashed red line; e.g., *R. exulans*; adapted from (*167*); CC BY 2.0].

A useful framework for understanding the overall influence of humans on island evolutionary patterns is provided by the taxon cycle model (*6*, *111*, *112*) (see Fig. 3). According to this model of ecological and evolutionary dynamics of insular lineages, islands are repeatedly colonized by generalist species, which become more specialized, locally differentiated, and geographically localized over time before eventually going extinct. Anthropogenic factors influence this cycle in two main ways (Fig. 3). First, the final stages of the cycle are often accelerated because of increased extinction rates for the specialized endemic forms, such as the extinction of (i) many of the bird species that were part of the Hawaiian honeycreeper/finch adaptive radiation (*111*, *113*), and (ii) island mammals that underwent the most pronounced body size shifts predicted by the island rule (Fig. 3) (*20*). Examples of increased vulnerability in extant stage 4 endemics include the tamaraw (*Bubalus mindorensis*), a critically endangered dwarf buffalo endemic to the mountainous interior of Mindoro Island in the Philippines (*114*). Second, the cycle is “restarted” by the anthropogenic introduction of new generalist species to islands, which over time can then progress through the different stages of the cycle. For example, kiore (Polynesian rat; *Rattus exulans*) populations introduced to Oceania and Wallacea have evolved larger body sizes relative to their mainland conspecifics (*107*), and those on the islands of New Zealand experienced range contractions caused by competitive interactions with other introduced commensals, such as black rats (*Rattus rattus*) and brown rats (*Rattus norvegicus*) (*115*).

## ANTHROPOGENIC IMPACTS ON ISLAND SPECIES INTERACTION PATTERNS

Island studies have played an important role in shaping our understanding of species interactions. Celebrated examples include studies highlighting the linkages between bill shapes of Darwin’s finches and the food they consume (*116*), and the coevolution of the Malagasy giant hawk moth (*Xanthopan praedicta*) proboscis and Star-of-Bethlehem orchids [*Angraecum sesquipedale* (*117*)]—an interaction famously predicted by Charles Darwin. At the community level, interactions are often studied through networks of interdependencies among species, such as between plants and pollinators (*118*). Island species interaction networks differ from their mainland counterparts in generally being relatively species poor, especially with respect to animal mutualists (*119*, *120*), comprising a higher proportion of generalist species (*120*, *121*), and involving weaker trait-matching between plant and animal mutualists (*122*). However, the extent to which these differences have emerged entirely through natural eco-evolutionary processes (*121*) or may have been accentuated by anthropogenic impacts on islands (*122*, *123*) is an open question. Of the patterns examined in this review, those concerning interaction networks are the most challenging to interpret in terms of human impacts. This is due to the (i) inherent difficulties in inferring interactions involving extinct species, and (ii) more general challenges associated with the study of interaction networks, including incomplete network sampling, the context dependence of interactions and associated considerations when comparing network properties across studies, and the argument that a clear theoretical link between various network metrics and ecosystem function is lacking (*124*).

Failing to account for anthropogenic extinctions and introductions when studying island interaction networks may result in incorrect inferences regarding natural (i.e., prehuman) network structure on islands (*125*). Together, these processes have caused substantial changes to the size and structure of island networks, their ecological function, metacommunity dynamics, spatial patterns, and evolutionary pressures. In terms of specific network patterns, anthropogenic extinctions and species introductions may result in researchers misinterpreting (i) modularity (i.e., the strength of division of a network into modules or subgroups), if non-native species have formed distinct novel interaction modules or entire modules are lost following extinctions; (ii) nestedness (i.e., the extent to which specialist species only interact with a subset of those species with which generalist species interact), where extinctions and non-native species can, respectively, disrupt the hierarchical patterns within networks or increase network nestedness; (iii) the level of specialization, where the introduction of non-native generalists increases the overall level of generalization in interaction networks; (iv) network stability, where past extinctions mask coevolved mutualisms; and (v) interaction strength, where non-native species alter the frequency and intensity of interactions between species [(*126**–**130*); for a recent mainland example, see (*131*)].

**Fig. 4.**
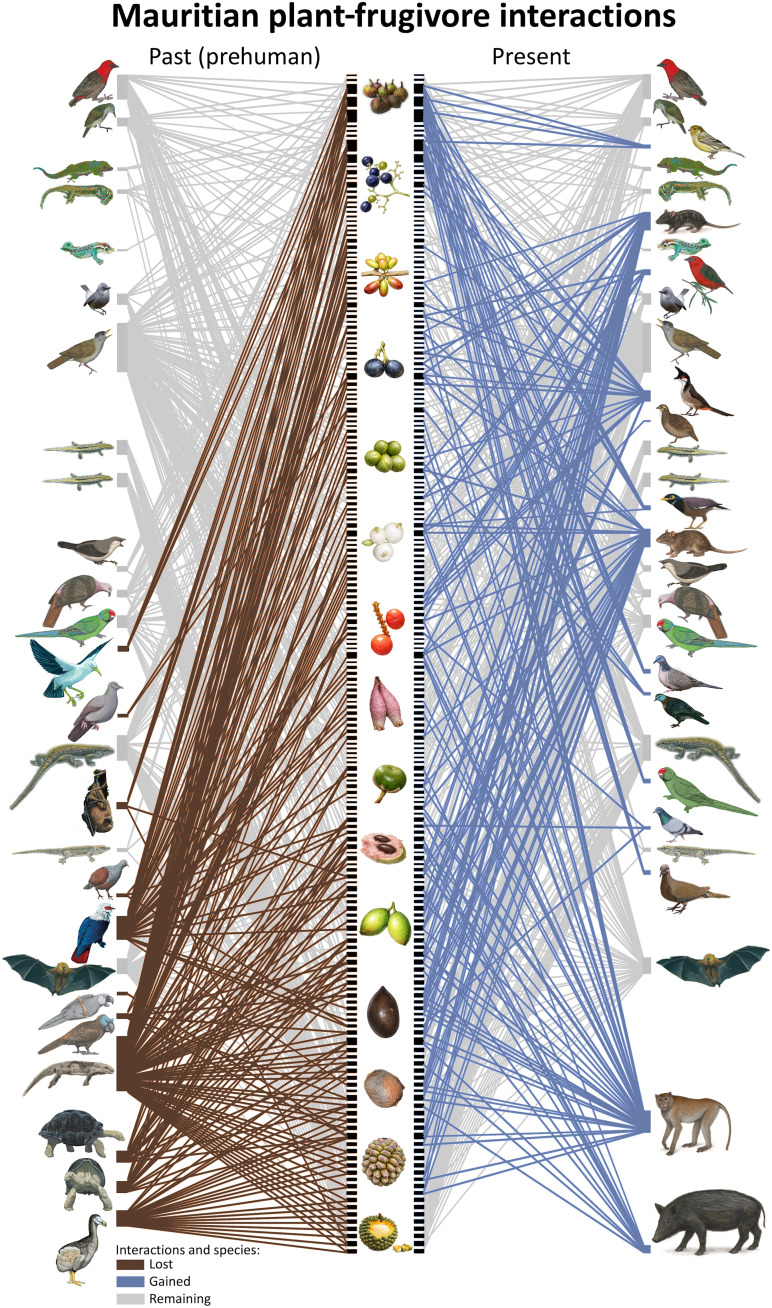
Changes in a plant-frugivore interaction network in response to anthropogenic extinctions and introductions. The two columns show changes to the Mauritian plant-frugivore interaction network from the prehuman period (i.e., before extinctions and introductions; left column) to the present day (right column) (based on 25% direct and 75% derived data). Extinct species and lost interactions in brown, introduced species and gained interactions in blue, and prehuman species and interactions that persist in gray [figure based on (*123*)]. Most of the large native frugivores have been lost, while introduced species include representatives of taxa not previously present (e.g., terrestrial mammals). Several interactions involving seed dispersers have been replaced by ecologically different interactions involving seed predators.

Beyond changes in network structure, extinctions and introductions can also systematically change underlying ecosystem functioning on islands (*46*, *123*, *132*). For example, extinctions have resulted in the loss of large numbers of native pollinators (e.g., the entire family Mohoidae on Hawai’i), seed dispersers (e.g., giant tortoises on various Indian Ocean islands), and predators [e.g., giant barn owls (*Tyto* spp.) on various Caribbean islands] (*10*, *123*). If non-native species provided like-for-like functional replacements of extirpated species, biases to our understanding of past island interactions would potentially be reduced. However, introductions not only involve (i) different taxa, thereby altering the taxonomic composition of interaction partners, but can also (ii) induce shifts in interactor behaviors, and (iii) lead to novel interaction pressures (e.g., predation by terrestrial mammals on islands previously lacking non-volant predators) (*13*, *133*, *134*), which alter the functional composition of island communities. For example, the replacement of native seed dispersers (e.g., flying foxes) with non-native seed predators (e.g., macaques) in Mauritius has led to a major shift in the functioning of the seed-dispersal network (Fig. 4) (*123*).

Species interactions play a key role in forming the spatial context of island ecology (*135*). However, large-bodied frugivores, including giant tortoises and many flightless birds, have been particularly affected by extinctions on many islands (*132*, *136*), leading to distinct shifts in both the local and the long-distance dispersal potential of large-seeded plants that rely on disperser traits such as large gapes, gut capacity, broad home ranges, and large energy reserves (*137*). The lost long-distance dispersers may once have formed multi-island, meta-community interaction networks (*138*), influencing gene flow, rescue effects, and migration histories among islands. More broadly, human disturbance has resulted in a patchwork of native, modified, and novel habitats on islands (*6*), erasing the ecological signatures that shaped natural spatial patterns of species occurrences and co-occurrences (e.g., some native plant species may no longer reproduce and disperse in areas where their native pollinators or seed-dispersers have been extirpated). Thus, it has become increasingly difficult to disentangle which interaction patterns in island ecosystems stem from natural eco-evolutionary processes and which from anthropogenic activities.

The severed and altered interactions that stem from anthropogenic extinctions and introductions represent previously unidentified eco-evolutionary selection pressures, highlighting links between altered interaction patterns and the natural evolutionary patterns covered in the previous section. The loss of specialized pollinators and seed dispersers, for example, may increase selection for more generalist flower and fruit phenotypes, as well as for wind dispersal (anemochory) to replace animal-mediated dispersal (zoochory). Phenotypic changes of this nature can subsequently lead to changes in trophic and competitive interactions (*134*, *139*, *140*), particularly if the traits involved have multiple effects or genetic links to other traits.

## CONCLUDING REMARKS

The long history of species extinctions and introductions on oceanic islands means that reconstructing the natural prehuman patterns of island biodiversity presents formidable challenges, to the extent that some authors have begun to question whether there is “a limit to the use of islands as ‘natural laboratories’” [(*32*), p. 82]. It could be argued that anthropogenic extinctions and introductions are leading oceanic islands—the primary focus of this review—to become more biotically similar to continental islands. Future work comparing the prehuman and contemporary biotas of oceanic and continental islands could provide interesting insights in this regard. There are nevertheless a number of promising approaches that can be used to better understand island biodiversity patterns and human impacts and, thus, address the Hookerian shortfall outlined in the introduction (*32*, *33*), including (i) using the information derived from the study of taxa with better fossil and historical records to provide insights for other taxonomic groups (*141*); (ii) the use of fossil pollen to track changes in plant composition through time (*9*); (iii) the extraction and analysis of ancient DNA [e.g., as has been undertaken for extinct Galápagos endemic snails (*142*)]; (iv) the increased use of herbarium and museum collections (*32*), as well as other types of historical data sources [e.g., travelers’ accounts and journals; (*143*)]; (v) the application of different analytical approaches that use various data sources—such as museum specimen data, expert knowledge, paleoecological sampling effort, and species discovery and observed extinction rates—to predict the number of unrecorded (“dark”) extinctions (*14*, *79*, *144*, *145*); and (vi) the development and application of mechanistic and simulation models to assess the effects of human impacts on biodiversity patterns (*146*). For several taxa, particularly birds, mammals, and plants, global databases of island extinctions and/or non-native species already exist (*10*, *61*, *97*, *147*, *148*). Thus, for these taxa, it is a case of expanding these databases when new knowledge becomes available and ensuring that they are used in relevant biodiversity analyses.

The primary focus of the present review has been on assessing the extent to which anthropogenic extinctions and introductions have influenced island biodiversity patterns. However, it is important to point out two additional consequences of human impacts on island biodiversity analyses. First, anthropogenic environmental change has also affected many of the environmental measures used as predictor variables in models of island biodiversity. A good example of this is island area. Area is an important predictor in most general models of island species richness (*149*). However, extensive native habitat loss on many islands (e.g., the Azores and the Pacific island of Rapa Nui have both lost more than 95% of their native forest cover) has reduced the effective area within which native species can survive (*6*, *82*), potentially weakening the explanatory power of island area in species richness models. Second, the extirpation, population reduction and expansion, and translocation of species in continental areas have modified mainland source pools, influencing the species that are available to colonize a given island (*74*, *150*). Given modern humans colonized many islands relatively late in human history (*6*), often long after the onset of the impacts of modern humans and other hominins on biodiversity on nearby continents (*61*, *151*), it is possible that these impacts could have influenced colonization dynamics on islands even before modern humans reached those islands.

Recent work has suggested that global extinction rates have slowed over the past ∼100 years (*152*) due, for example, to the possibility that the most extinction-prone species have already been lost and the success of conservation programmes [see (*6*) for a review in an island context]. While an intriguing prospect, we remain cautious given the reliance of such analyses on IUCN Red List data, which do not consider pre–1500 CE extinctions (*14*), and which have known taxonomic biases (e.g., we have a better idea of the number of island bird extinctions than island beetle extinctions), an issue acknowledged in (*152*). This is particularly important when we consider the extinction of species before they have been described [so-called dark or “cryptic” extinctions; (*98*, *145*)], of which there may have been many in certain relatively understudied island taxa. For example, while the role of invasive predatory snail species of the genus *Euglandina*, first introduced to the Hawaiian Islands in 1955, as an extinction driver of large endemic land snail species on several islands in the Pacific is relatively well documented, their impact on, and the resultant extinction of, small endemic species is “completely unknown but might be as important” (*17*). Notwithstanding the apparent variation in the rate of recent global species extinction described in (*152*), it is evident that islands have provided a disproportionate number of anthropogenic extinctions in recent centuries, and the magnitude of previous extinctions and extirpations on many islands is such that, even if island extinctions ceased today, the alterations to biodiversity patterns would already be substantial.

As outlined above, our review has mainly focused on human impacts to biodiversity patterns on oceanic islands and some well-studied continental fragments (e.g., Madagascar). Reviewing the literature in this field has revealed two research gaps in regard to island type. First, there is an urgent need to better understand the impacts of humans on biodiversity patterns on other types of islands, particularly continental-shelf islands. Second, much of the work in this area has focused on a subset of well-studied oceanic archipelagos (e.g., Hawai’i), leaving some parts of the world relatively understudied. In particular, there are relatively few relevant studies incorporating islands in Southeast Asia [exceptions include (*20*, *69*)]. Southeast Asia comprises thousands of islands, many of which support large numbers of endemic species but differ from the islands in the aforementioned well-studied archipelagos in that many (i) have existed for tens of millions of years, (ii) have had relatively complex geological histories (e.g., are of composite origin and/or involving periods of connectivity with continental land masses), and (iii) have had human presence for tens to hundreds of thousands of years [e.g., archaic humans are believed to have reached the island of Flores over a million years ago, with modern humans arriving ∼50,000 years ago; (*153*)]. Despite this, and with some exceptions (*20*), they (iv) have typically experienced relatively few known global anthropogenic extinctions [e.g., (*154*)]. The latter could be a true pattern [e.g., the presence of numerous islands in relatively close proximity could increase regional connectivity and act to lower extinction rates; (*154*)] or could be due to a lack of fossil data. The distribution of known extinctions strongly points toward the lack of fossils as at least part of the explanation since a majority of known mammal extinctions from the region of Wallacea come from the single well-studied island of Flores (*61*). However, even if we discount global extinctions, we know that humans have had impacts on the biota of many Southeast Asian islands—including island-level extirpations and ancient inter-island translocation events—and thus the region represents a priority for future research into island biodiversity patterns in the Anthropocene.

Ultimately, contemporary patterns of island diversity, distinctiveness, and distribution emerge from the combined effects of both classic biogeographic drivers and the more recent anthropogenic factors including those of trade and land use change, with economic area (based on the amount of developed land) and economic isolation/connectivity (as measured by international trade routes and tourism) increasingly driving biodiversity patterns on islands as globalization proceeds (*23*, *32*, *44*, *54*). Thus, contemporary biodiversity patterns may still be suitable for analysis and the development of predictive models provided that any approach includes assessments of the intensity and geographic dynamics of anthropogenic as well as natural processes. Such an integrative approach seems essential if we are to reconstruct the natural (prehumanity) patterns of biological diversity across the world’s islands and develop effective strategies for conserving or recovering the natural patterns as the Anthropocene progresses.
